# Chemical Mechanical Lapping of 316 Based on Solid-Phase Fenton Reaction

**DOI:** 10.3390/ma19112200

**Published:** 2026-05-23

**Authors:** Luguang Guo, Kangyi Zhou, Yaxin Tian, Zongding Bao, Li-An Zhang, Jiahuan Wang, Tianchen Zhao

**Affiliations:** 1 College of Mechanical Engineering, Quzhou University, Quzhou 324000, China; glg7127@163.com (L.G.); huangdizky@outlook.com (K.Z.); 18235355947@163.com (Y.T.); zhaotianchen1989@126.com (T.Z.); 2 Zhejiang Key Laboratory of Intelligent Manufacturing for Aerodynamic Equipment, Quzhou University, Quzhou 324000, China; 3 Ultra-Precision Machining Center, Key Laboratory of Special Purpose Equipment and Advanced Processing Technology, Ministry of Education and Zhejiang Province, Zhejiang University of Technology, Hangzhou 310023, China; wangjiahuan@zjut.edu.cn; 4 Zhejiang Haina Semiconductor Co., Ltd., Quzhou 324000, China; zhanglian@hainasemi.cn; 5 School of Mechanical Engineering, Zhejiang University, Hangzhou 310058, China

**Keywords:** chemical mechanical lapping, solid-phase Fenton reaction, AISI 316, high-performance abrasive tool

## Abstract

**Highlights:**

- A solid-phase Fenton chemo-mechanical lapping method was developed for AISI 316 stainless steel.
- SF-CML achieved high-efficiency and low surface roughness removal by coupling Fe_3_O_4_-containing tools with H_2_O_2_ slurry.
- The optimum condition was pH 2.5 with 0.05 wt% H_2_O_2_, giving 16.64 μm/min MRR and 20.95 nm Sa.
- Potentiodynamic polarization curves and XPS results confirmed an oxidation-assisted removal mechanism dominated by Fe oxidation.
- SF-CML outperformed conventional Fenton CMP and mechanical lapping in balancing efficiency and surface quality.

**Abstract:**

To achieve both a high material removal rate and excellent surface quality, this paper proposes a solid-phase Fenton chemo-mechanical lapping (SF-CML) method. Using high-purity type 316 stainless-steel as the research object, a solid lapping tool containing Fe_3_O_4_ microparticles was employed in synergy with an H_2_O_2_-based slurry. Under locally high-pressure and high-temperature conditions, Fe^2+^ ions are released, which in turn catalyze the generation of highly reactive hydroxyl radicals (·OH). These radicals promote the formation of an oxide layer on the workpiece surface, which is continuously removed through mechanical action. The results show that at pH 2.5 and an H_2_O_2_ concentration of 0.05 wt%, SF-CML achieves the best processing performance, with an MRR of 16.64 μm/min and a Sa as low as 20.95 nm. XPS, EPR, and other characterization methods collectively provided evidence for the oxidation of the sample surface and the existence of ferrous ions and hydroxyl radicals in the slurry, thereby confirming the effectiveness of the solid-phase Fenton reaction. Compared with conventional homogeneous Fenton CMP and pure mechanical lapping, SF-CML not only significantly improves removal efficiency but also effectively enhances surface quality. This method avoids the problems of easy precipitation and low removal efficiency commonly encountered in traditional homogeneous Fenton systems, providing a new technical pathway for high-efficiency precision processing of metallic materials.

## 1. Introduction

Ultra-precision machining has increasingly become a critical enabling technology for the development of advanced manufacturing systems as modern science and technology continue to progress. Technologies such as ultra-precision cutting [1,2,3,4,5], ultra-precision grinding [6,7,8,9,10], flexible polishing [11,12,13,14], and precision non-traditional machining [15,16,17] are increasingly pursuing high material removal efficiency, with the scale of material removal approaching the atomic level [18].

Chemical mechanical polishing (CMP) generates a passivation layer or reaction layer on the surface material through chemical reactions, such as oxidation and dissolution, with the components in the polishing slurry [19]. Subsequently, this reaction layer is removed by the mechanical abrasion action of abrasive particles in the slurry and the polishing pad. The dynamic equilibrium and synergistic interplay between ‘chemical’ and ‘mechanical’ processes enable CMP to effectively overcome the limitations inherent in either pure chemical etching or pure mechanical lapping with machining precision and surface integrity, thereby achieving atomic-level planarization of the sample surface. Qi et al. proposed an ammonium hexafluorophosphate (NH_4_PF_6_) assisted CMP process for indium phosphide (InP). By exploiting the hydrolysis reaction of In^3+^ to achieve a material removal rate of 424 nm/min, while attaining a root mean square roughness (Sq) of 0.0862 nm [20]. Song et al. integrated the ReaxFF molecular dynamics (ReaxFF MD), the Gaussian mixture model (GMM), and the crowed porcupine optimization algorithm (CPO) to propose an atomic level surface integrity modeling method for CMP, which provides a theoretical foundation for the development of ultra-precision machining of polycrystalline diamond with low damage [21]. Liu et al. investigated the synergistic removal mechanisms of different abrasives and oxidizers during the CMP of polycrystalline aluminum nitride (AlN), revealing that S_2_O_8_^2−^ exhibited the highest adsorption energy on the AlN (001) surface, thereby facilitating the formation of Al-O bonds and promoting oxidative removal. Experimental results obtained with the optimized oxidizer produced an ultra-smooth surface with Sq < 0.18 nm; however, the material removal rate was not improved and remained at approximately 2.1 μm/h [22].

To reconcile the high material removal rate of ultra-precision grinding with the high quality of chemical mechanical polishing, Zhou proposed chemo-mechanical grinding (CMG) [23]. Wu used a grinding wheel embedded with Cr_2_O_3_ abrasive particles to process sapphire substrates via CMG, achieving near damage-free machining under optimized process conditions. The maximum material removal rate reached nearly 500 μm/h; however, under damage-free processing conditions, the highest removal rate was only 0.8 μm/h [24]. Zhang et al. investigated the material removal mechanism of silicon wafers during the CMG process and found that stress release associated with the fracture of soft abrasives could effectively suppress surface and subsurface damage [25]. The main limitation of CMG is that its high removal efficiency depends on the forced feed imposed by the grinding machine, and subsurface damage remains unavoidable when large grinding depths are employed.

Wu et al. developed a novel environmentally friendly slurry for stainless steel, achieving a material removal rate (MRR) of 82.14 nm/min and a surface roughness (Sa) of 0.286 nm in CMP [26]. Lee et al. performed CMP on 304 stainless steel using a slurry containing 39 wt% abrasives, 1 wt% oxalic acid, and 0.03 wt% H_2_O_2_ at pH 1.5 for 3 min, and reported an increase in surface roughness of 4 nm [27]. Chen et al. employed a Fenton-type polishing slurry composed of H_2_O_2_ and ferric chloride for 304 stainless steel, achieving an MRR of 700 nm/min and a surface roughness better than 5 nm [28]. In summary, current research on CMP of difficult-to-machine materials has primarily focused on the development of novel polishing slurries, with efforts directed toward either improving the material removal rate or further enhancing surface quality. However, the removal efficiency of conventional CMP remains limited due to its use of free abrasives.

This study proposes a Fenton-like method based on solid-phase oxidants, which achieves high-efficiency material removal and low surface roughness of metallic materials under low contact pressure through the combined action of a solid-phase oxygen catalyst and an oxidant-containing polishing slurry. High-purity type 316 stainless-steel was used as the workpiece material. Based on the literature review, Luo et al. recently employed an approach of micro-nano bubbles enhanced immersed CMP (MNBEICMP) for orthopedic implant of 316 stainless steel, in which the material removal rate was improved through the cavitation effect, reaching 1.421 μm/h [29]. This reported result is used as a reference for comparison in the following sections. This paper aims to evaluate the effectiveness of the SF-CML process in achieving high-efficiency material removal for 316 stainless steel. By investigating the chemical-mechanical synergy between solid-phase Fenton oxidation and abrasive action, this work seeks to validate the catalytic mechanism of the tool surface and its impact on MRR. The characterization of subsurface crystalline integrity and residual resistance remains a subject for future comprehensive investigation.

## 2. Solid-Phase Fenton Reaction Mechanism and Experimental Details

### 2.1. Material Removal Mechanism of SF-CML

**Figure 1:**
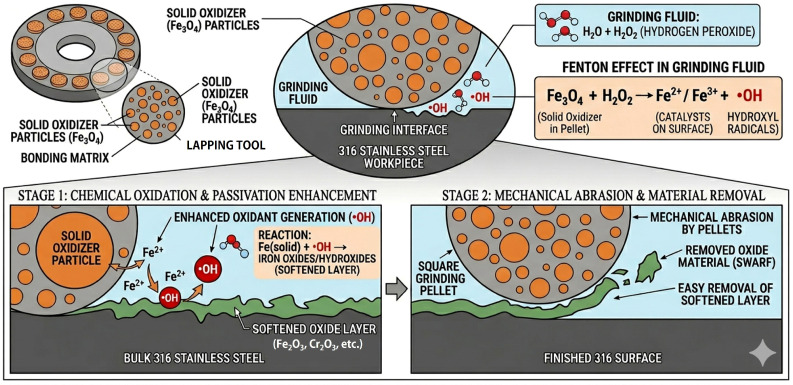
Material removal mechanism based on solid-phase Fenton reaction of AISI 316.

The principle of the solid-phase Fenton chemo-mechanical lapping method is illustrated in Figure 1. The lapping wheel contains Fe_3_O_4_ microparticles. Fe^2+^ is released under localized high-temperature conditions [30]. The released Fe^2+^ reacts with H_2_O_2_ or H_2_O in the grinding slurry to generate a large amount of hydroxyl radicals (·OH) with strong oxidative activity, together with a certain amount of hydroxide ions (-OH). Under acidic conditions, -OH is neutralized, while ·OH oxidatively attacks the 316 stainless steel surface to form an oxide layer, which is subsequently removed by the mechanical lapping action of the lapping tool. By eliminating the use of liquid-phase Fe^2+^, this method can sustain efficient material removal while avoiding precipitation issues arising from poor slurry compatibility [31,32].

### 2.2. Experimental Setup

The lapping wheel used in this study was a self-developed diamond lapping tool containing solid Fe_3_O_4_ micropowder. The formulation of the raw materials is listed in Table 1, where the additives mainly consisted of pore-forming agents and lubricants. The lapping tool was compacted under a pressure of 500 MPa. After compaction, the lapping tool was bonded onto the hard disk in a staggered arrangement, and the surface flattening was subsequently performed using a high-precision grinding machine (SLG 80, Schneider GmbH & Co.KG, Fronhausen, Germany). After each test, the tool was dressed using a green silicon carbide plate (#80) to maintain optimal tool performance. The resulting tool exhibited a uniform distribution of iron oxide microparticles without visible aggregation or macro-defects. The surface maintained its structural integrity throughout the whole lapping process.

**Figure 2:**
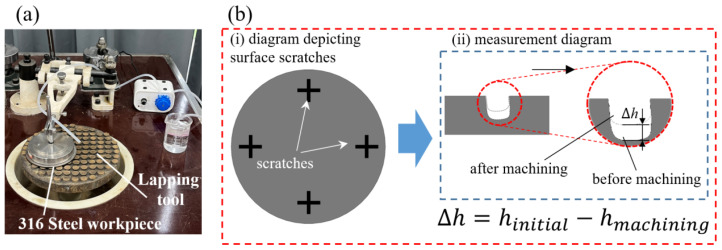
Experimental setup: (**a**) processing system, (**b**) principle of removal rate measurement.

SF-CML experiments were performed using a three-axis polishing machine (Xibin Optoelectronics Co., Ltd., Wuxi, China). The experimental processing system is shown in Figure 2a. To ensure process reproducibility, kinematic and mechanical parameters were strictly regulated as summarized in Table 2. A constant lapping load of 70 N was applied, while the rotational speed of the lapping tool was maintained at 60 rpm. The workpiece was driven by the differential between internal and external frictional forces, ensuring both processing uniformity and a consistent relative velocity. The slurry was continuously delivered to the center of the tool via a peristaltic pump at a stable flow rate of approximately 10 mL/min. It should be noted that different processing times were selected for SF-CML (1 min) and conventional Fenton CMP (10 min) based on their distinct reaction kinetics. The measurement method for the material removal rate is illustrated in Figure 2b. The test samples were 316 stainless steel disks with a diameter of 100 mm. The sample surface was rough polished with W20 Al_2_O_3_ first, to obtain an initial surface roughness Sa of approximately 50 nm. Subsequently, uniform-depth grooves were generated on the sample surface by laser engraving. An optical profilometer (ultra-depth of field microscopy, VHX-2000C Digital Microscope, Keyence Corporation, Osaka, Japan) was employed to measure the change in groove depth (∆h) before and after lapping. To ensure metrological rigor, each sample was measured at five different locations (center and four quadrants) to account for local topography variations. The material removal rate under different processing conditions was calculated. The corresponding equation is given in Equation (1).

(1) $$MRR=\frac{∆h(\mu m)}{t\left(min\right)}$$

In Equation (1), *t* denotes the lapping time. In the investigation of the effects of the chemical environment and oxidant concentration on processing quality, the lapping time was fixed at 1 min, considering the relatively high material removal rate of the fixed lapping tool. Each test was repeated three times, and the average value was taken as the final MRR.

The surface quality of the samples at different processing stages was characterized by white light interferometry (Super View W1, Chotest Technology Inc., Shenzhen, China) and ultra-depth of field microscopy. XPS (ESCALAB 250Xi, Thermo Fisher Scientific, Waltham, MA, USA) analysis was performed to investigate the changes in the valence states of surface elements. As no graphite phase was present in the samples, the binding energy was corrected by referencing the C 1s peak to 284.8 eV. Before XPS investigation, the samples were cleaned ultrasonically in anhydrous ethanol and deionized water for 3 min to ensure the removal of any physically adsorbed debris.

An electrochemical workstation (CHI660F, Shanghai Chenhua Instruments Co., Ltd., Shanghai, China) was employed to investigate the corrosion pathway of steel during the SF-CML experiments. The potentiodynamic polarization curves were obtained in the potential range from −0.5 to 0.9 V vs. OCP. To ensure the reproducibility of the measurements, the samples were first maintained at open circuit for 10 min before the polarization tests.

In the comparative experiments presented in the final section, Fenton CMP was conducted using a polishing cloth (Zhejiang Planary Electronic Materials Co., Ltd., Quzhou, China) as the polishing pad, with a processing duration of 10 min to ensure that the processing limit was approached. In the mechanical lapping experiments, a diamond lapping plate without the solid oxidant was used, while the other components remained the same as those in the SF-CML tool. The processing parameters were kept identical to those adopted in the SF-CML experiments.

## 3. Results and Discussion

### 3.1. Effect of pH Environment on Removal Quality and Efficiency

**Figure 3:**
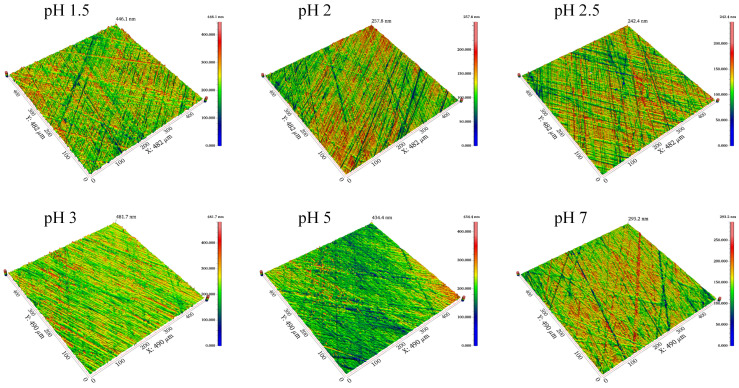
Variation in surface morphology with pH.

The evolution of surface morphology under different pH values is shown in Figure 3. At various pH values, all machined surfaces exhibit interlaced lapping marks, indicating that the material removal is dominated by plastic removal caused by mechanical ploughing. The roughness values show certain differences: under strongly acidic conditions (pH 1.5), the highest surface roughness reaches Sa 38.11 nm; at pH values from 2 to 7, the surface roughness Sa is stable below 30 nm, and the values are very close to each other. This behavior may be attributed to the effect of the strongly acidic environment on the lapping tool, which reduced the stability of the machining process.

**Figure 4:**
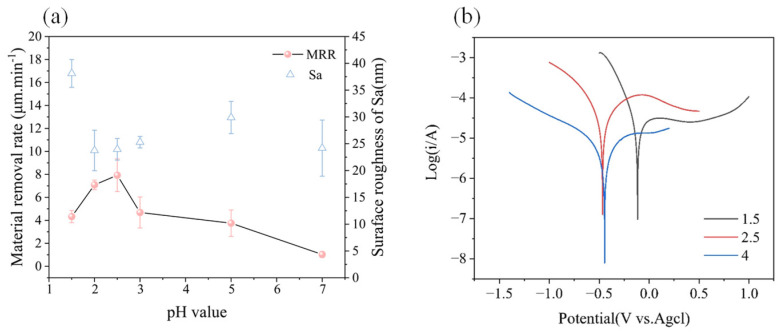
Variation in removal rate (**a**) and surface roughness (**b**) with pH.

The chemical environment has a significant influence on the performance of the oxidant. The evolution of the removal rate under different pH values is shown in Figure 4a. The highest removal rate of 7.92 μm/min is achieved at pH 2.5. As the pH increases, the removal rate gradually decreases, reaching 1.02 μm/min under neutral conditions (pH 7). Under strongly acidic conditions (pH 1.5), the removal rate also decreases. Two possible reasons are speculated: (1) the lapping tool is also oxidized in the strongly acidic environment, leading to a decrease in its strength and thus a reduction in removal efficiency; (2) 316 stainless steel sample forms a dense passivation layer in the strongly acidic environment, resulting in a decrease in removal efficiency.

To investigate the influence of chemical environment on the material removal mechanism, potentiodynamic polarization tests were conducted at three pH values (1.5, 2.5, and 5), with the results shown in Figure 4b. The cathodic branches of the three polarization curves exhibit similar slopes, indicating that the cathodic reduction mechanism remains largely unchanged. In the anodic region, the polarization curves show a clear active-to-passive transition.

As the pH increases, the current rises more slowly with increasing potential during the initial stage of anodic polarization, suggesting a gradual enhancement of the passivation ability. However, the corrosion current density does not show a simple positive correlation with the material removal rate (MRR). Although the highest corrosion current density (approximately −4.8 Log(i/A)) is observed at pH 1.5, the MRR at this pH is not the highest. This is because, under excessively acidic conditions, the surface may become over-activated or undergo uncontrolled dissolution, failing to form a coherent, brittle, and mechanically shearable chemically modified layer. Consequently, the abrasives cannot effectively remove the material [33].

In contrast, a moderate corrosion current density (approximately −6.8 Log(i/A)) is observed at pH 2.5. Under this condition, we infer that a thin and uniform oxide/hydroxide layer forms on the surface. This layer is chemically softened by the reactive species generated from the Fenton reaction, yet it retains sufficient mechanical integrity to be effectively removed by abrasives. At pH 2.5, the synergy between chemical softening and mechanical removal achieves an optimal balance. When the pH is increased to 5, the corrosion current density decreases to its lowest value (approximately −8.0 Log(i/A)). In this case, the passive film becomes too stable and dense to be disrupted mechanically, resulting in a low MRR.

In summary, the optimal processing condition does not correspond to the maximum corrosion current, but rather falls within a “synergistic window” of moderate electrochemical activity. In this experiment, pH 2.5 yields both the highest MRR and good surface quality; therefore, this condition was selected for subsequent experiments.

### 3.2. Effect of Oxidant Concentration on Removal Quality and Efficiency

**Figure 5:**
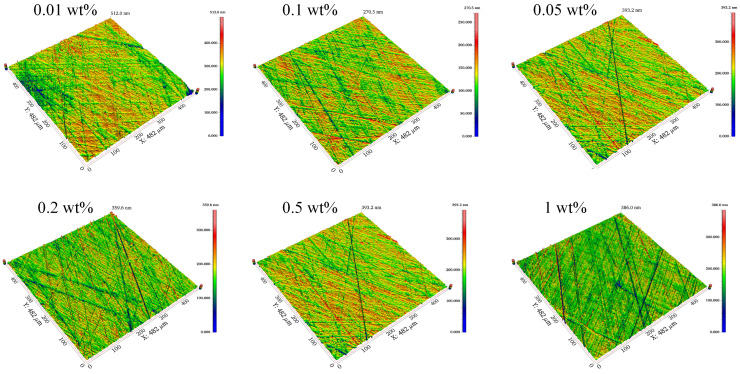
Variation in surface morphology of oxidant concentration.

The evolution of surface morphology under different oxidant concentrations is shown in Figure 5. At various oxidant concentrations, all machined surfaces still exhibit interlaced lapping marks, indicating that material removal is dominated by plastic removal caused by mechanical ploughing. However, the ploughing depth of the lapping marks becomes shallower, suggesting a further increase in the contribution of chemical action to material removal. In general, the roughness value first decreases and then increases with increasing oxidant concentration, further demonstrating that as the oxidation effect intensifies, the proportion of chemical removal increases.

Under an almost oxidant-free condition (0.01 wt%), the surface roughness Sa was 27.25 nm, indicating that the oxidation effect was negligible at such a low oxidant concentration. In the range of 0.05 wt% to 0.1 wt%, the roughness reached its lowest values, remaining at approximately 20 nm, due to the removal of the uniformly distributed oxidation-modified layer. With further increase in oxidant concentration, the roughness gradually rose and stabilized at 30 nm, because the oxidation led to the formation of a dense passivation layer, resulting in deteriorated surface quality.

**Figure 6:**
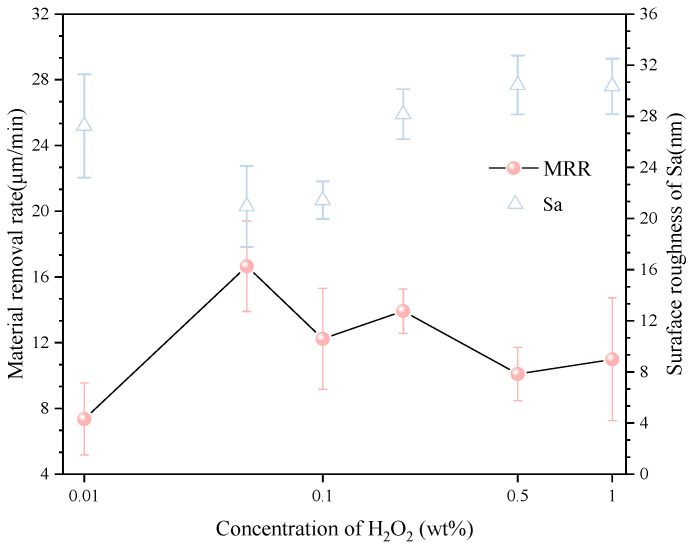
Variation in removal rate and surface roughness of oxidant concentration.

The oxidation effect and its mechanism directly affect the material removal efficiency. The evolution of the removal rate under different oxidant concentrations is shown in Figure 6. At an oxidant concentration of 0.01 wt%, the removal rate was 7.35 μm/min, which is essentially consistent with that under oxidant-free conditions, indicating that no oxidation effect occurred. In the concentration range from 0.05 wt% to 0.5 wt%, the removal rate slowly decreased with increasing oxidant concentration, which is attributed to the formation of a dense oxide film on the surface that hindered material removal. Furthermore, in this stage, the removal rate stabilized at approximately 10 μm/min, indicating that the compactness of the oxide film reached its peak, and the oxidant concentration no longer affected the removal efficiency.

Considering that at an oxidant concentration of 0.05 wt%, the material removal rate reached its highest value (16.64 μm/min) while the surface roughness achieved its lowest value (Sa 20.95 nm), it is concluded that under the components of the lapping tool used in this experiment, pH 2.5 and an H_2_O_2_ concentration of 0.05 wt% constitute the optimal process level. It is specifically noted that if subsequent researchers change the lapping tool components or process conditions, new process experiments must be conducted to determine the optimal process level.

### 3.3. Analysis of Oxidation Mechanism During the SF-CML

**Figure 7:**
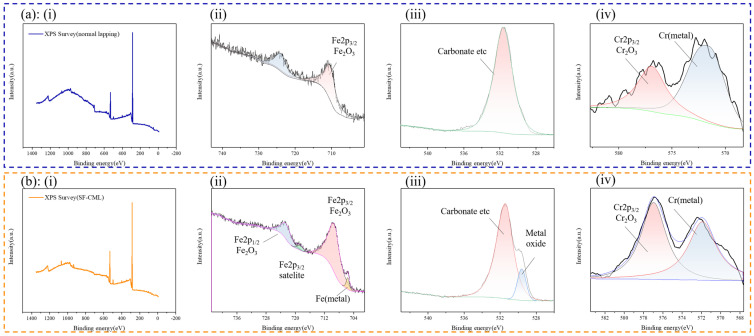
Comparison of AISI 316 surface state prepared by different lapping methods: (**a**) traditional mechanical lapping; (**b**) SF-CML. Label (**i**) shows the total XPS survey spectrum of the processed sample, while (**ii**) to (**iv**) display the high-resolution spectra of Fe 2p, O 1s, and Cr 2p, respectively.

To clarify the mechanism of the solid-phase Fenton lapping method, the surface chemical states of 316 stainless steel after conventional lapping (Figure 7a) and SF-CML (Figure 7b) were systematically compared using XPS.

Comparing the Fe2p spectra, the conventionally lapped surface (Figure 7a(ii)) shows a predominant metallic state with weak oxidation signals. In contrast, the SF-CML processed surface (Figure 7b(ii)) exhibits significantly enhanced Fe^3+^ peaks. Parallel results are observed in the Cr2p spectra. For the conventional surface (Figure 7a(iv)), the Cr signal corresponds to the intrinsic thin passive film. However, after SF-CML (Figure 7b(iv)), the Cr^3+^ characteristic peak (~576.8 eV) is markedly intensified. This indicates that the hydroxyl radicals (·OH) produced by the Fenton reaction do not solely react with Fe; they also effectively attack and oxidize the Cr-rich components within the passive film.

Interestingly, even after the intense Fenton reaction in SF-CML, metallic Fe and Cr signals are still detectable. This is a critical physical observation: it proves that the SF-CML process is not a simple chemical etching process. Instead, it is a dynamic “oxidation-stripping” cycle. The Fenton reaction “softens” the surface by transforming the dense, protective passive film into a more voluminous and less adherent mixed Fe-Cr oxide layer. This sacrificial layer is then rapidly removed by the mechanical action of the abrasives, re-exposing the metallic substrate for the next oxidation cycle.

The O1s spectra further support this transition. The SF-CML surface (Figure 7b(iii)) shows a prominent lattice oxygen O^2−^ peak, which is reasonably assigned to the complex (Fe, Cr)_x_O_y_ species formed during the process. These findings confirm that the presence of Fe_3_O_4_ on the tool surface maintains high catalytic stability, ensuring a continuous supply of ·OH to destabilize the Cr-rich passive structure, thereby significantly increasing the material removal rate. To further quantify the impact of the Fenton reaction on the 316 stainless steel surface, high-resolution Fe2p and Cr2p spectra were deconvoluted to compare the atomic percentages of different valence states.

(1) Evolution of the Cr-rich passive film

The analysis of Cr2p spectra reveals a significant shift in the oxidation state of the surface. For the conventional lapping sample, the ratio of metallic chromium (Cr) to oxidized chromium (Cr^3+^) was 64.82% to 35.18%, reflecting the presence of a stable, thin native passive film. In contrast, after SF-CML treatment, the proportion of Cr^3+^ notably increased to 53.43%, while Cr decreased to 46.57%. This quantitative increase in Cr^3+^ provides direct evidence that the hydroxyl radicals (·OH) generated via the Fenton effect successfully penetrate and destabilize the original Cr-rich passive layer, converting the metallic substrate into a thicker, less protective oxide matrix.

(2) Enhanced Fe oxidation and material removal

The Fe2p spectra further distinguish the two processes. On the conventionally lapped surface, the total Fe signal intensity was relatively low, indicating a surface still dominated by the Cr-rich passivation layer that inhibits extensive Fe oxidation. However, the SF-CML surface exhibited a robust Fe signal, with a quantified Fe to Fe^3+^ ratio of 94.39% to 5.61%. While Fe (metal) remains dominant due to the continuous mechanical removal of the oxide layer, the presence of clear Fe^3+^ peaks—coupled with the high MRR—confirms the rapid and continuous formation of Fe-based oxidation products. The detection of significant metallic Fe (94.39%) alongside these oxides proves that the mechanical stripping action of the abrasives is highly efficient, constantly re-exposing the fresh metal surface to the Fenton reagents.

The consistent enhancement of MRR and the characteristic XPS peaks of Fe-based oxidation products suggest that the Fe_3_O_4_ particles on the tool surface remained catalytically active and stable during the SF-CML process.

**Figure 8:**
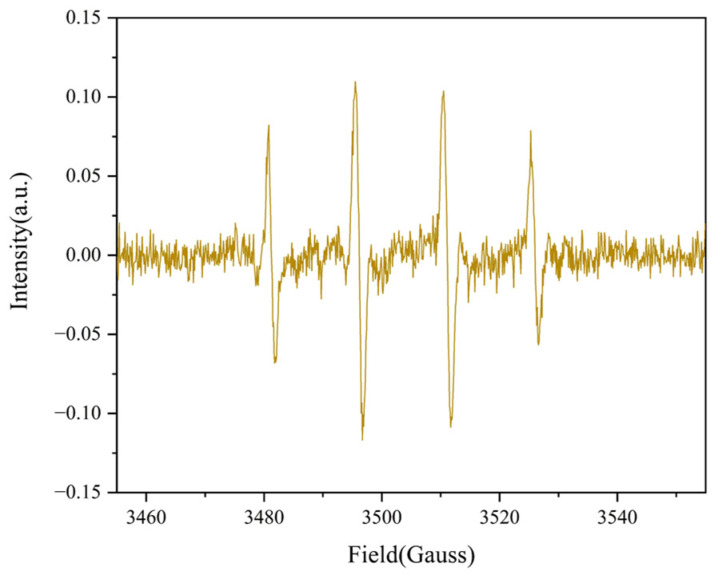
Detection of hydroxyl radicals in the slurry.

The synergy between mechanical action and chemical oxidation is fundamental to the high-efficiency polishing of 316 stainless steel. ICP-OES analysis of the lapping fluid revealed an iron concentration of 184.58 mg/L. This confirms that under the acidic environment (pH 2.5) and mechanical friction, Fe_3_O_4_ particles in the solid-phase tool undergo localized dissolution, releasing active iron species into the interface. The catalytic activity of these released ions was further verified by EPR spectroscopy. As shown in Figure 8, the detection of a distinctive 1:2:2:1 quartet signal (hyperfine coupling constant approx. 14.9 G) confirms the generation of ·OH via the Fenton reaction. The combination of ICP-OES and EPR data provides a complete experimental chain: the tool-released iron ions effectively decompose H_2_O_2_ to produce ·OH at the contact points. For 316 stainless steel, these highly reactive ·OH radicals can penetrate the dense Cr_2_O_3_-rich passivation layer, converting it into a more hydrated, porous, and mechanically weaker oxide/hydroxide layer, which is then efficiently removed by the abrasives.

## 4. Comparative Experiment

**Figure 9:**
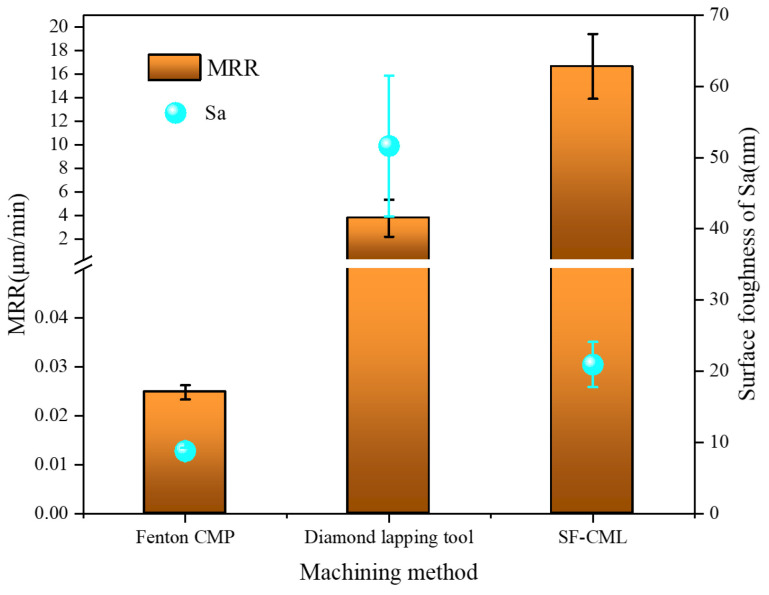
Material removal rate and surface quality of different machining methods.

**Figure 10:**
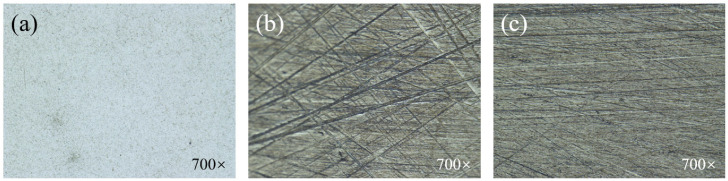
Comparison of surface quality of different polishing processes: (**a**) homogeneous Fenton CMP, (**b**) without solid oxidant, (**c**) SF-CML.

Figure 9 and Figure 10 present a comparison of the surface quality and material removal rate of 316 stainless steel after processing by different precision machining methods. Conventional Fenton CMP achieved the best surface quality, with the surface roughness Sa lower than 10 nm and a smooth surface, as shown in Figure 10a. Mechanical lapping results in a surface roughness of approximately 50 nm, with numerous scratches on the surface (Figure 10b). For the sample processed by SF-CML, the scratch depth is significantly reduced compared to mechanical lapping, and the surface roughness reaches about 20 nm (Figure 10c).

In contrast to the trend observed for surface quality, Fenton CMP exhibits the lowest removal rate, only about 0.02 μm/min, which is close to the value reported in Ref. [24]. For comparison, the removal rate of mechanical lapping reaches approximately 4 μm/min, while that of SF-CML is as high as 16.64 μm/min. Considering that the rotational speed and lapping pressure are the main process factors affecting the removal rate in mechanical lapping, it can be concluded that compared with mechanical lapping, SF-CML achieves a significant improvement in both material removal rate and surface quality. As a pre-polishing step before CMP, SF-CML can greatly reduce the amount of material removal required in the subsequent polishing step, thereby improving overall processing efficiency.

## 5. Conclusions

In this study, a chemical mechanical lapping method based on the solid-phase Fenton reaction (SF-CML) is proposed to achieve high-efficiency and low roughness material removal of metallic materials. Using high-purity type 316 stainless-steel as the research object, the effects of pH value and H_2_O_2_ concentration on the processing performance were systematically investigated. The results show that:

1. The optimal process conditions are pH 2.5 and an H_2_O_2_ concentration of 0.05 wt%. Under these conditions, the material removal rate reaches a maximum of 16.64 μm/min, and the surface roughness (Sa) attains a minimum of 20.95 nm.
2. Potentiodynamic polarization curves indicate that the corrosion current density is positively correlated with the removal rate, confirming the promoting effect of electrochemical activity on material removal. XPS analysis further reveals that the Fe^3+^ content on the surface after SF-CML processing is significantly higher than that after conventional lapping, verifying an oxidation dominated removal mechanism.
3. Compared with conventional homogeneous Fenton CMP and pure mechanical lapping, SF-CML achieves a better balance between removal efficiency and surface quality.

While the SF-CML process yields a significantly higher MRR and a smooth surface finish, it should be noted that the current study focuses on the material removal kinetics and the chemical evolution of the surface. Further specialized characterizations, such as cross-sectional TEM or EBSD, would be required to fully quantify the sub-surface state and removal mechanism, which is beyond the scope of this initial validation of the SF-CML mechanism.

## Figures and Tables

**Table 1:** Components of the solid-phase Fenton lapping tool.

Components	Value
Copper powder	0.665
Diamond 3000#	0.19
Resin binder	0.095
Fe_3_O_4_ micropowder (50 μm)	0.05
Other additives	Balance

**Table 2:** Key processing parameters of the SF-CML experiments.

Parameter	Value
Sample size	Φ100 mm × 3 mm
Lapping tool diameter	300 mm
Applied pressure	8.9 kPa
Rotational speed	60 rpm
Slurry flow rate	10 mL/min
Lapping duration (SF-CML)	1 min
Lapping duration (Fenton CMP)	10 min
Ambient temperature	25 °C

## Data Availability

The original contributions presented in this study are included in the article. Further inquiries can be directed to the corresponding author.

## References

[1] Sun L.H., Chen M.H., He T., Yan H.Z., To S., Wu Y.B., Yip W.S. (2024). Enhancing ductile regime ultra-precision diamond turning of curved zinc selenide (ZnSe) optics by using straight-nosed diamond tools with cutting-edge-slipping. J. Manuf. Process..

[2] Das A., Singh C., Bajpai V., Chandravanshi M.L. (2025). Feasibility of cost-effective ultra-precision diamond turning using ceramic bearing spindle. Int. J. Adv. Manuf. Technol..

[3] Cao Y., Zhao X.S., Li G., Zong W.J., Sun T. (2023). Study regarding the influence of process conditions on the surface topography during ultra-precision turning. J. Manuf. Process..

[4] Lee W.B., Cheung C.F., Li J.G., To S., Du J.J., Yin Z.Q. (2007). Development of a virtual machining and inspection system for ultra-precision diamond turning. Proc. Inst. Mech. Eng. Part B-J. Eng. Manuf..

[5] Pei L., Wu H.B. (2019). Effect of ultrasonic vibration on ultra-precision diamond turning of Ti6Al4V. Int. J. Adv. Manuf. Technol..

[6] Wei Z.Z., Li C., Wang Z.Y., Zakharov O., Yan G.Y., Li M.F., Geng Y. (2026). A comprehensive dynamic force model involved in grinding of B4C ceramics. Int. J. Extrem. Manuf..

[7] Li C., Wang K.C., Zakharov O., Cui H.L., Wu M.T., Zhao T.C., Yan Y., Geng Y. (2025). Damage evolution mechanism and low-damage grinding technology of silicon carbide ceramics. Int. J. Extrem. Manuf..

[8] Zhang Y., Wu T., Wang Y.F., Hu B., Li C. (2026). Adaptive nonlinear-error control method for five-axis machining of complex structures. Int. J. Mech. Sci..

[9] Wu T., Zhang Y., Wang Y.F., Hu B., Li C. (2026). An error-controlled G3-continuous oriented toolpath optimization algorithm and modified speed planning for five-axis machining. J. Manuf. Process..

[10] Li C., Piao Y.C., Meng B.B., Hu Y.X., Li L.Q., Zhang F.H. (2022). Phase transition and plastic deformation mechanisms induced by self-rotating grinding of GaN single crystals. Int. J. Mach. Tools Manuf..

[11] Chen H.Y., Hong B.B., Wu M.X., Wang L., Lyu B., Yang Y., Yao Y., Chen C., Chen P., Beri T.H. (2025). A novel method of liquid film shearing polishing for achieving high-quality and low-damage polycrystalline tungsten. J. Manuf. Process..

[12] Chen H.Y., Wang L., Peng F., Shen M.M., Hang W., Beri T.H., Zhang H., Zhao J., Han Y., Lv B. (2025). Efficient chemical mechanical polishing of W promoted by Fenton-like reaction between Cu^2+^ and H_2_O_2_. Trans. Nonferr. Met. Soc. China.

[13] Yang G.L., Wang Y.T., Zhu Y.Y., Chang L., Tong K.K., Li Y.N., Wang Y., Jia S., Zhu H., Zhao T. (2026). Grain engineering for low-temperature Cu/SiO_2_ hybrid bonding: A microstructure-driven thermal expansion model. Mater. Charact..

[14] Farghal A.F., Mansoure T.H., Mohamed M.G., Mohammed A.A.K., Kuo S.W. (2026). Effect of carbonyl group position on redox-active anthraquinone-, phenanthrenequinone-, and benzil-linked hexaazatrinaphthalene conjugated microporous polymers for supercapacitor electrodes. Chem. Eng. J..

[15] Zhou J.J., Wang J.H., Chen X.Q., Wang X., Wang J.H., Dai Z.H., Yuan J. (2026). Research on single-crystal diamond surface polishing process and material removal mechanism based on inductively coupled plasma etching reactions. Diam. Relat. Mat..

[16] Han Y.X., Luo C.Y., Li X.L., Wang X., Wang J.H., Yuan J.L. (2026). Insights into the atomic-scale removal mechanism of SiC in plasma-assisted polishing. Appl. Surf. Sci..

[17] Khan M., Zhou T.F., Yu Q., He Y.P., Yang X.Z., Guo W.J., Ahmad H. (2026). A single step laser activation strategy for high-integrity electroless Ni-P plating on binderless WC. Opt. Laser Technol..

[18] Tian H.L., Yan S.H., Sun Z.Z., Wang H.C., Yan H.P. (2024). Effect of nano-scratch speed on removal behavior of single crystal silicon. Diam. Abras. Eng..

[19] Wurz M.C., Pape F., Wark M., Gatzen H.H. (2011). Investigation of Liquid Additives on the Nano-Hardness of NiFe during Polishing. Tribol. Online.

[20] Fu S.G., Zhou J.W., Wang C.W., Ge Y.C., Luo C., Qi Y.H. (2026). One-Step, High-Removal-Rate and Low-Damage Chemical Mechanical Polishing of InP Enabled by Hydrolysis Activated PF6-with In Situ Fluoride Passivation. Adv. Sci..

[21] Song Z., Guo X.G., Zhang W.X., Deng Y.M., Li Z.Z., Fan G.H., Kang R., Wang X. (2025). Atomic-scale surface integrity prediction of chemical mechanical polishing polycrystalline diamond: Insights from ReaxFF MD and CPO-GMM-LSSVM model. Diam. Relat. Mat..

[22] Liu S.T., Zhang B.G., Xian W.H., Wang Y.J., Qin S.H., Liu Y. (2026). Mechanistic insights into high-efficiency polishing of AlN single crystals: Synergistic effects of abrasives and oxidants. Appl. Surf. Sci..

[23] Gao S., Huang H., Zhu X.L., Kang R.K. (2017). Surface integrity and removal mechanism of silicon wafers in chemo-mechanical grinding using a newly developed soft abrasive grinding wheel. Mater. Sci. Semicond. Process.

[24] Wu K., Zhou L.B., Shimizu J., Onuki T., Yamamoto T., Ojima H., Yuan J. (2017). Study on the potential of chemo-mechanical-grinding (CMG) process of sapphire wafer. Int. J. Adv. Manuf. Technol..

[25] Zhang Y., Kang R.K., Ren J.W., Lang H.Y., Gao S. (2023). Mechanical effect of abrasives on silicon surface in chemo-mechanical grinding. Int. J. Mech. Sci..

[26] Wu Y.W., Wang D., Zhang Z.Y., Zhao F., Zhou H.X., Liu X.Q., Yang X. (2025). A close atomic surface of stainless steel produced by novel green chemical mechanical polishing using silica and lanthana mixed abrasives. Nanoscale.

[27] Lee D., Kim H., Pak B., Kim D., Jeong H., Lee H. (2017). Electrochemical Analysis of the Slurry Composition for Chemical Mechanical Polishing of Flexible Stainless-Steel Substrates. J. Frict. Wear.

[28] Chen J.P., Peng Y.A., Wang Z.K., Lv F.G. (2024). Influence of Fenton-like reactions between hydrogen peroxide and ferric chloride on chemical mechanical polishing 304 stainless steel. Int. J. Adv. Manuf. Technol..

[29] Luo Z., Zhang Z.Y., Zhou H.X., Yang M., Wang Y.M., Li S.L., Shao J., Zhang X., Liu Z. (2026). A novel approach of micro-nano bubbles enhanced immersed chemical mechanical polishing on orthope dic implants with curve d surface using a developed polisher. J. Mater. Sci. Technol..

[30] Horng J.H., Jeng Y.R., Chen C.L. (2004). A model for temperature rise of polishing process considering effects of polishing pad and abrasive. J. Tribol.-Trans. ASME.

[31] Zhang J., Yang T., Wang H.Y., Yang K., Fang C., Lv B., Yang X. (2015). Study on treating old landfill leachate by Ultrasound-Fenton oxidation combined with MAP chemical precipitation. Chem. Speciat. Bioavailab..

[32] Jafari A.J., Golbaz S., Kalantary R.R. (2013). Treatment of hexavalent chromium by using a combined Fenton and chemical precipitation process. J. Water Reuse Desalin..

[33] Wang J.H., Lyu B.H., Jiang L., Shao Q., Deng C.B., Zhou Y.F., Wang J., Yuan J. (2021). Chemistry enhanced shear thickening polishing of Ti-6Al-4V. Precis. Eng. J. Int. Soc. Precis. Eng. Nanotechnol..

